# Seroprevalence of West Nile virus among blood donors in mainland France, 2021 to 2022

**DOI:** 10.2807/1560-7917.ES.2026.31.26.2500808

**Published:** 2026-07-02

**Authors:** Pauline Jourdan, Karine Barthélémy, Nadège Brisbarre, Christine Isnard, Pierre Gallian, Stéphane Priet, Xavier de Lamballerie

**Affiliations:** 1 Unité des Virus Émergents (UVE: Aix-Marseille Univ, Università di Corsica, IRD 190, Inserm 1207, IRBA), Marseille, France; 2 Établissement Français du Sang Provence Alpes Côte d'Azur et Corse, Marseille, France; 3 Établissement Français du Sang, La Plaine Saint-Denis, France

**Keywords:** West Nile Virus, seroprevalence, France, blood donors, risk factors

## Abstract

**BACKGROUND:**

West Nile virus (WNV) is an orthoflavivirus maintained in an enzootic cycle between birds and ornithophilic *Culex* mosquitoes. Human infections are usually asymptomatic or paucisymptomatic, while severe neurological disease occurs mainly in children, elderly and immunocompromised individuals. Despite evidence of WNV circulation in France since the 1960s, nationwide seroprevalence data are unavailable.

**AIM:**

We wanted to determine the seroprevalence of WNV among blood donors in mainland France to characterise the epidemiology of WNV and assess risk factors and potential implications for transfusion safety and public health.

**METHODS:**

Sera from 44,490 volunteer blood donors collected in 2021–2022 in France were screened for anti-WNV IgG by ELISA on pools of up to four samples, with samples from non-negative pools tested individually. ELISAs for various flaviviruses and virus neutralisation tests were used to assess non-negative results.

**RESULTS:**

Seroprevalence was low overall, similar in pools and individual samples (0.87% and 0.97%, respectively). Prevalence was 1.13% in Nouvelle-Aquitaine and 1.81% in Ile-de-France, preceding the first local human cases reported in 2023 and 2025 in these regions, respectively. The risk factors identified as being associated with WNV seropositivity were geographic (living in the south of France: Occitanie, Provence-Alpes-Côte d'Azur and Corsica) and related to ABO blood group.

**CONCLUSION:**

Seroprevalence of West Nile virus in France is low but variable, suggesting that WNV may have circulated undetected in some areas. Monitoring flavivirus prevalence in blood donors can serve as an early warning system for human infections and provide valuable data for public health preparedness.

**Key public health message**

**What did you want to address in this study and why?**

West Nile virus (WNV) can cause severe neurological disease in humans. In recent years, France has reported increasing numbers of WNV cases, including in regions with no previous history of virus detection. We therefore wanted to estimate, the prevalence of WNV among blood donors in mainland France, including geographical distribution and risk factors of exposure to WNV, to assess potential implications for transfusion safety and public health.

**What have we learnt from this study?**

After analysing 44,490 sera from voluntary blood donors collected in 2021–2022, we found a low overall national seroprevalence of WNV (0.97%). However, we also identified signals that WNV had been spreading silently in some unexpected areas, beyond the regions already known to be affected and several associated risk factors including region of residence and blood group.

**What are the implications of your findings for public health?**

Our findings support the need to extend WNV surveillance in humans, mosquito vectors and other accidental hosts (mainly horses and birds) beyond the known affected areas, including monitoring products of human origin (blood and organs).

## Introduction

West Nile virus (WNV) is an emerging neurotropic mosquito-borne virus of the genus *Orthoflavivirus* (formerly classified within the genus *Flavivirus* following recent International Committee on Taxonomy of Viruses (ICTV) taxonomic updates) belonging to the Japanese encephalitis complex [1]. This virus persists in the environment through an enzootic cycle that involves Passeriformes (sparrows and corvids), Charadriiformes (gulls and auks) and Strigiformes (owls) as reservoirs and ornithophilic mosquitoes (*Culex* species) as vectors [2-4]. *Culex* mosquitoes are endemic to France, and their activity period extends from June to late November, meaning that most WNV cases occur in summer or autumn. West Nile virus can be transmitted to accidental dead-end hosts, such as humans, horses or dogs [5] through the bite of an infected mosquito [6]. Occasionally, other less common transmission routes have also been reported, such as nosocomial transmission through blood transfusions [7] and organ transplantations [8], breastfeeding or the transplacental route during pregnancy [9].

West Nile virus is known to be asymptomatic in most cases but can occasionally cause a wide range of symptoms, ranging from influenza-like illness to serious neurological disorders such as meningitis, neuropathies and encephalitis [10]. In France, WNV was first identified in 1962 in the Camargue region (south-east France) in a horse with meningoencephalitis [11]. Subsequently, numerous cases and deaths among horses were reported in southern France in the 2000s [12]. In humans, cases of neurological disorders linked to WNV were described in 2003 in Fréjus (south-east France), where seven human cases of WNV infection were reported, including three cases of encephalitis and four cases of febrile illness [13]. In France, nucleic acid testing (NAT) for WNV is performed selectively in regions where autochthonous human cases are detected to ensure blood transfusion safety [14].

Human autochthonous cases of WNV infection were recently identified outside the Mediterranean region. Between May and November 2023, 29 locally acquired human cases were reported in the Nouvelle-Aquitaine region (Atlantic south-west coast) [15]. In 2025, three human cases of WNV were identified in the Île-de-France region (Paris region), two of whom developed severe neurological disorders [16,17].

To date, no nationwide seroprevalence data have been available in France. The most comprehensive recent study was performed between 2016 and 2020 by testing human, dog and horse samples in the Camargue area and the city of Montpellier (both in the Mediterranean region), reporting a WNV seroprevalence of 1.25% [18].

Serological studies of WNV are complicated by antibody cross-reactivity among flaviviruses due to conserved epitopes on the envelope protein. In France, this is particularly relevant for Usutu virus (USUV), which cocirculates with WNV, and for tick-borne encephalitis virus (TBEV), which is endemic and for which vaccination is available. Dengue virus (DENV), while not endemic in mainland France, is frequently imported through travel and represents an additional source of cross-reactive antibodies.

We conducted a large-scale seroprevalence study, analysing 44,490 blood donor sera samples from France collected by national blood banks across all mainland departments in 2021–2022. Since WNV shares antigenic similarities with other flaviviruses, we developed a confirmatory algorithm to distinguish true WNV infections from potential cross-reactive serological responses to DENV, TBEV and USUV, and to improve the specificity of seroprevalence estimates. This study provides the first nationwide assessment of WNV seroprevalence in France, offering important insights into WNV circulation in the French population, epidemiological risks, and blood donation safety.

## Materials and methods

### Sample collection

This study used a preexisting sample collection obtained from the EpiArboTiq study (Etude de séroprévalence du virus TBE dans la population des donneurs de sang en France et la constitution de la collection). The samples were collected and managed by the French national blood bank (EFS) and came from healthy volunteers aged 18 to 70 years. All participating donors were considered eligible after a standard medical interview (regulatory medical screening) and provided informed consent for their samples to be used for seroprevalence studies. No specific selection was applied beyond the standard eligibility criteria of the national blood service; therefore, the sampling was consecutive, and only one sample per donor was included in the study. The donations selected for the study were chosen to reflect the proportion of the population living in rural and urban areas within the metropolitan French departments. In addition, recruitment bias was minimised by excluding donations conducted in school settings (to avoid age-related bias) and donations involving military personnel (to avoid sex-related bias).

A total of 44,490 serum samples, collected between November 2021 and June 2022 were available for this study, covering 95 of the 96 metropolitan French departments (the Creuse department was not included; Supplementary Table S1 present the numbers of samples by department and administrative regions).

### Data collection

Sociodemographic data were collected alongside blood samples. Further details on the data collected are available in Supplementary Table S2.

### ELISA on pooled and individual samples

**Figure 1:**
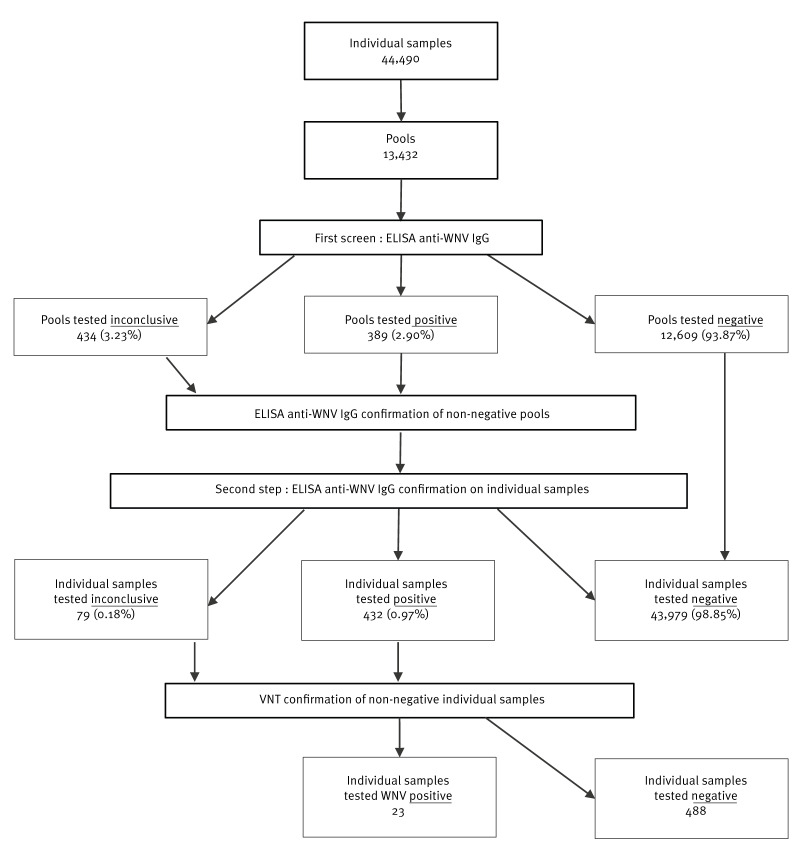
Flowchart of West Nile virus IgG screening and confirmation, mainland France, November 2021–June 2022

VNT: virus neutralisation test; WNV: West Nile virus.

To preserve assay sensitivity, the pooling protocol was optimised to increase the final serum concentration, resulting in a dilution series ranging from 1:100 for single samples (manufacturer’s protocol) to 1:32.5 for 4-sample pools. Validation in a 700-sample subset demonstrated that the pooling method had the same sensitivity as individual testing (see Figure 1; the pooling methodology is described in the Supplementary Methods). A total of 44,490 individual samples were automatically pooled, yielding 13,432 pools grouped by department for analysis (Figure 1).

All pools underwent initial screening using the commercial Anti-West Nile Virus ELISA (IgG) kit (Euroimmun, Lubeck, Germany) targeting the WNV envelope protein. Samples were classified based on the ratio as negative (< 0.7), inconclusive (0.7 ≤ ratio < 1.1) or positive (≥ 1.1). Samples from reactive (both inconclusive and positive) pools were then tested individually using the commercial IgG ELISA kits (Euroimmun): Anti-Dengue Virus Type 1–4 ELISA (IgG) and Anti-Usutu Virus ELISA (IgG) that target the envelope proteins, Anti-TBE Virus ELISA (IgG) that uses inactivated virus particles (strain K23) as antigens and Anti-West Nile Virus ELISA (IgG).

### Virus neutralisation test

Viral strains used for virus neutralisation testing were provided by the European Virus Archive (EVAg) repository (https://www.european-virus-archive.com/): dengue virus 2 (001v-EVA928), tick-borne encephalitis virus (001v-EVA134), Usutu virus (001v-EVA138) and West Nile virus (001v-EVA140). Details of the virus neutralisation test (VNT), including the cell lines used, multiplicity of infection (MOI) and cytopathic effect (CPE) read-out, are provided in the Virus neutralisation test section of the Supplementary Methods.

All anti-WNV IgG ELISA non-negative samples were confirmed by VNT against a panel of flaviviruses (DENV, TBEV, USUV WNV), as previously described [19,20]. A detailed protocol is described in the Supplementary Methods. A titre ≥ 20 was considered a positive result.

### Algorithm for sample classification

We developed an algorithm to assign a final serostatus for WNV to each sample, taking into account serological cross-reactivity among flaviviruses. This confirmatory algorithm categorises samples into seven distinct statuses based on ELISA results and virus neutralisation titre values against DENV, TBEV, USUV and WNV. It was designed to compare the strength of the serological response to WNV with responses to the other viruses to exclude results likely attributable to cross-reactivity (Box).

Box **Sample category serostatus for blood samples containing West Nile virus based on ELISA results**

**Negative (NEG)**

Samples with a WNV ELISA ratio < 0.8 and a WNV VNT titre of 10.

**Confirmed (CONF1, CONF2 and CONF3)**

In CONF1, the WNV VNT titre was > 10 and at least twice the VNT titres of DENV, TBEV and USUV.

CONF2 included samples not classified as CONF1, with a WNV VNT titre > 10 and DENV, TBEV and USUV VNT titres of 10.

CONF3 included samples not belonging to CONF1 or CONF2, with a WNV ELISA ratio > 1.1 and at least twice the ELISA ratio of DENV, TBEV and USUV along with a WNV VNT titre > 10 and equal to or greater than the VNT titres of DENV, TBEV and USUV.

**Probable**

Samples not belonging to the CONF1, CONF2 or CONF3 groups, with a WNV ELISA ratio > 1.1 and higher than the DENV, TBEV and USUV ELISA ratio and a WNV VNT titre ≥ the VNT titres of DENV, TBEV, USUV.

**Possible**

This was assigned to samples when no other status could be applied, the WNV ratio was ≥ 0.8 and 0.75 times greater than the DENV, TBEV and USUV ELISA ratios.

**Other**

Included samples that did not belong to any other status but with a WNV ELISA ratio > 0.8 (presumably mostly samples with antibodies against other flaviviruses).

**Presumed positive**

For the final analysis, we grouped the confirmed and probable statuses into a single category of presumed positive samples.

CONF: confirmed; DENV: dengue virus; TBEV: tick-borne encephalitis virus; USUV: Usutu virus; VNT: virus neutralisation test; WNV: West Nile virus.Further details are provided in Supplementary Table S3, which summarises the classification criteria.

Algorithm thresholds (e.g., ELISA positivity cut-offs, ≥ 2-fold differences in ELISA ratios or VNT titres) were selected based on manufacturer recommendations, established practices in flavivirus serology and internal assay validation to balance sensitivity and specificity in a low-prevalence setting where no single gold standard exists for past WNV infection.

### Map construction

The seroprevalence maps were created using the free, open source QGIS software version 3.40.5 Bratislava (www.qgis.org). The datasets for the French metropolitan departments and regions were obtained from https://www.data.gouv.fr/fr/datasets/carte-des-departements-2-1/ and https://www.data.gouv.fr/fr/datasets/contours-des-regions-francaises-sur-openstreetmap/ accessed on 15 April 2025.

### Risk factor analysis

Risk factor analysis was performed using R version 4.5.0 (R Foundation, Vienna, Austria) in RStudio version 2025.05.1. Univariate associations between potential risk factors and WNV serological status were first explored using Fisher’s exact test. Variables included age group (18–20, 21–30, 31–40, 41–50, 51–60 and ≥ 60 years), sex (male/female), region of residence, living environment (urban, semiurban or rural), ABO blood group and selected medical history variables (meningitis, encephalitis or radiculitis). Living environment was classified based on the self-reported driving time to the nearest supermarket (urban: <15 minutes by car, semi-urban: 15–30 minutes by car and rural: >30 minutes by car). Multivariate analysis was conducted using a binomial logistic regression model (glm function).

All variables considered epidemiologically relevant and available in the dataset were included in the model and no automated variable selection procedure (e.g., stepwise or backward elimination) was applied. This approach was chosen to minimise data-driven selection bias and to allow estimation of adjusted odds ratios (aOR) while accounting for potential confounding effects. Adjusted odds ratios and 95% confidence intervals (CI) were calculated. Univariate analyses were considered exploratory and used primarily to describe crude associations; therefore, no formal correction for multiple testing was applied. Interpretation of risk factors was primarily based on the results of the multivariate model. Statistical significance was defined as p < 0.05.

## Results

### Study population

Among the 44,490 blood donors included in the study, sex distribution was balanced (males 50.1% (22,277/44,490); females 49.9% (22,213/44,490)). The study population was distributed across age groups as follows: 5% were 18–20 years, 16% were 21–30, 18% were 31–40, 22% were 41–50, 22% were 51–60, and 17% were 60 years or older.

### ELISA based seroprevalence of West Nile virus

During initial screening by anti-WNV ELISA, 823 (6.12%) of the 13,432 pools were non-negative (positive or inconclusive), of which 389 (2.90%) were positive (Figure 1). This corresponded to a pool-based seroprevalence estimate of 0.87% (95% CI: 0.79–0.96) (assuming that no pool contained more than one positive sample - see Supplementary Methods for the calculation). After testing all samples included in the non-negative pools, we identified 432 individual positive samples among the 44,490 donations, yielding a refined estimate of the national seroprevalence at 0.97% (95% CI: 0.88–1.06). Validation of the pooling strategy in a 700-sample subset showed a very strong correlation between individual-sample and pooled-sample ELISA ratios (R^2^ = 0.9741). Supplementary Figure S1 presents the correlation between the ELISA ratio of pooled and individual samples. Furthermore, seroprevalence estimates derived from pooled testing were highly consistent with those obtained from individual testing at the department level (R^2^ = 0.8892). Supplementary Figure S3 presents the correlation between WNV seroprevalences obtained from pooled and individual samples at the department level.

**Figure 2:**
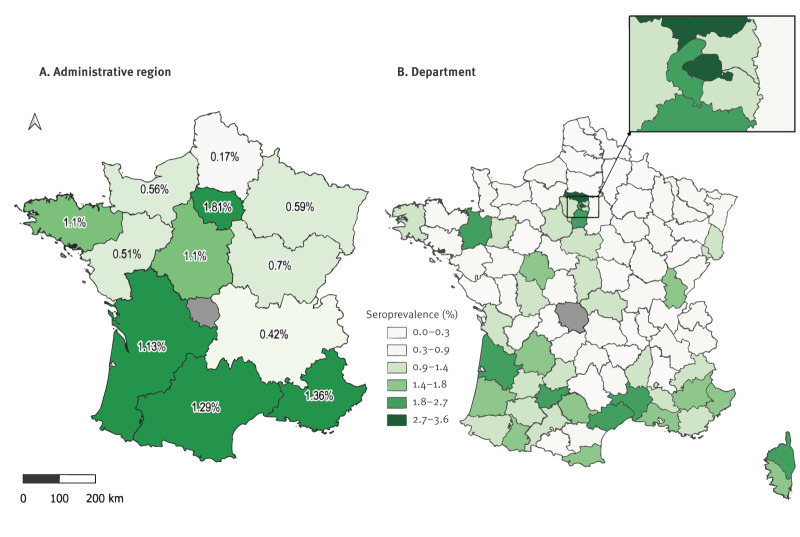
Seroprevalence of anti-West Nile virus IgG-positive samples by (A) administrative region and (B) department, mainland France, November 2021–June 2022

The spatial distribution of the positive samples was mapped across the French administrative regions and departments (Figure 2). Supplementary Figure S2 presents a map showing the seroprevalence of anti-WNV IgG positive pooled samples across administrative regions and departments. Classification of samples was performed using the algorithm described in Supplementary Table S4. Prevalence varied geographically, with the highest values observed in southern France, particularly in Corsica (1.95% by pool testing; 2.12% by individual testing), the Mediterranean regions of Provence-Alpes-Côte d'Azur (1.36% by pool testing; 1.36% by individual testing) and Occitanie (1.17% by pool testing; 1.29% by individual testing) and along the Atlantic south-west coast in the Nouvelle-Aquitaine region (0.87% by pool testing; 1.13% by individual testing). Unexpectedly elevated prevalence was also noted in the Île-de-France region, and to a lesser extent in the Centre and Bretagne regions. At the department level, prevalence ranged from 0% in some areas to 2.29% (pool testing) and 2.45% (individual testing) in Corsica. Departments bordering the Mediterranean, Corsica, Île-de-France and the Atlantic south-west coast regions showed the highest seroprevalence. Several inland departments between these coastal regions demonstrated comparable prevalence values. Results from both testing strategies were highly correlated and consistent (Supplementary Figure S3 presents the correlation between WNV seroprevalences obtained from pooled and individual samples at the department level).

The Creuse department (in grey) was not evaluated.

### Confirmatory algorithm-based West Nile virus seroprevalence

**Figure 3:**
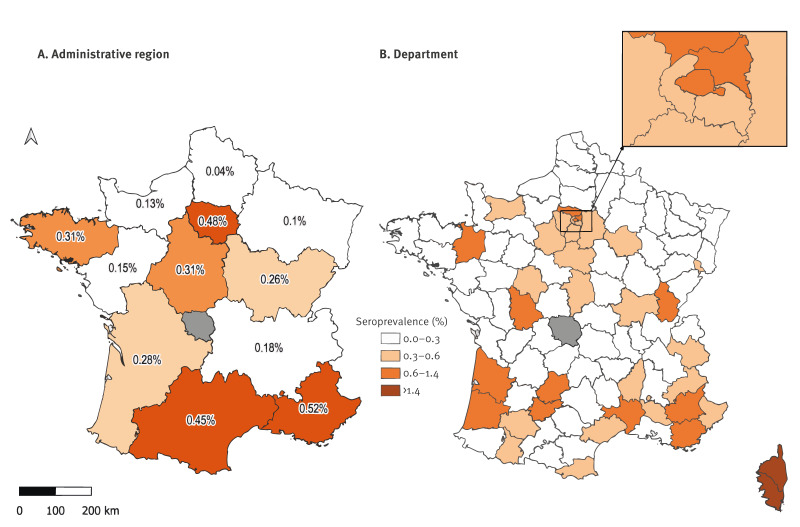
Seroprevalence of West Nile virus-positive samples, as defined by the confirmatory algorithm, across (A) administrative region and (B) department, mainland France, November 2021–June 2022

Applying the confirmatory algorithm refined the spatial analysis (Figure 3; classification of samples was performed using the algorithm described in Supplementary Table S4) and established a lower, more accurate nationwide seroprevalence of 0.31% (95% CI: 0.26–0.37). The anticipated high seroprevalence was confirmed in the Mediterranean regions of Provence-Alpes-Côte d'Azur, Occitanie and Corsica Island, where values ranged from 0.45% to 1.53%. The elevated prevalence previously noted in the Île-de-France region (0.48%) was also confirmed at a level comparable to that of the Provence-Alpes-Côte d'Azur and Occitanie regions. The confirmation algorithm also identified several non-Mediterranean departments with elevated seroprevalence, including Gironde (0.76%), Landes (0.78%) and Vienne (0.76%) on the Atlantic coast and Ille-et-Vilaine (0.91%) in the Bretagne region. Several inland departments located between these coastal regions also demonstrated comparable prevalence values, including Lot (0.83%) and Tarn-et-Garonne (1.09%) in the Occitanie region, Jura (0.76%) in the Bourgogne-Franche-Comté region and Paris (0.65%), Seine-Saint-Denis (0.65%) and Val-d’Oise (0.65%) in the Île-de-France region.

The Creuse department (in grey) was not evaluated.

### Geographic distribution of West Nile virus seroprevalence

**Figure 4:**
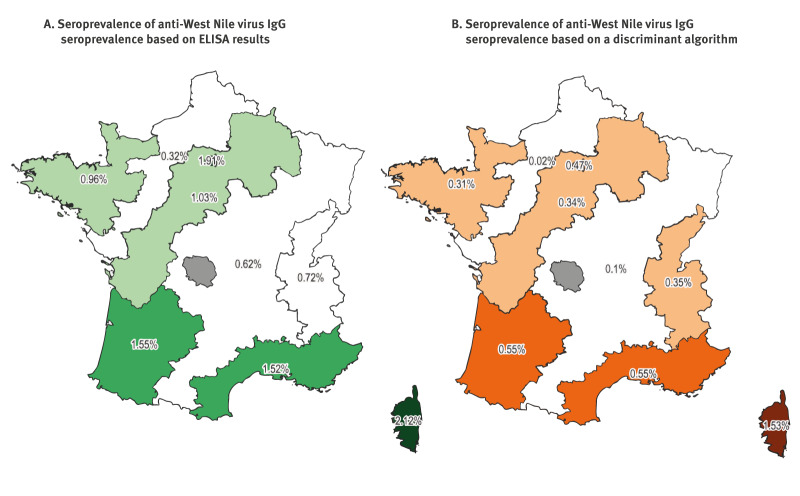
Seroprevalence of anti-West Nile virus IgG seroprevalence based on (A) ELISA results and (B) a discriminant algorithm, represented by customised areas, mainland France, November 2021–June 2022

To visualise the main areas of WNV circulation in France, departments were first grouped according to the lowest observed seroprevalence values, allowing the identification of background areas with minimal circulation. From this baseline, departments were subsequently aggregated into progressively higher seroprevalence categories based on both initial ELISA results and the confirmatory algorithm (Figure 4). This stepwise spatial grouping revealed two corridors with background seroprevalence values. As expected, three high-prevalence areas were confirmed: Corsica Island, the Mediterranean coast and the Atlantic south-west coast, with the highest values ranging from 0.55% to 1.53%. A distinct corridor of intermediate seroprevalence (0.34%) was also identified between the two background corridors, encompassing the Île-de-France region (0.47%) and exhibiting seroprevalence comparable to the Bretagne region (0.31%) and the Alps (0.35%).

The prevalence values derived from the detection of anti-WNV IgG in individual samples by ELISA (A) in individual samples after applying a confirmatory algorithm (B) was mapped.

### Risk factors analysis

**Figure 5:**
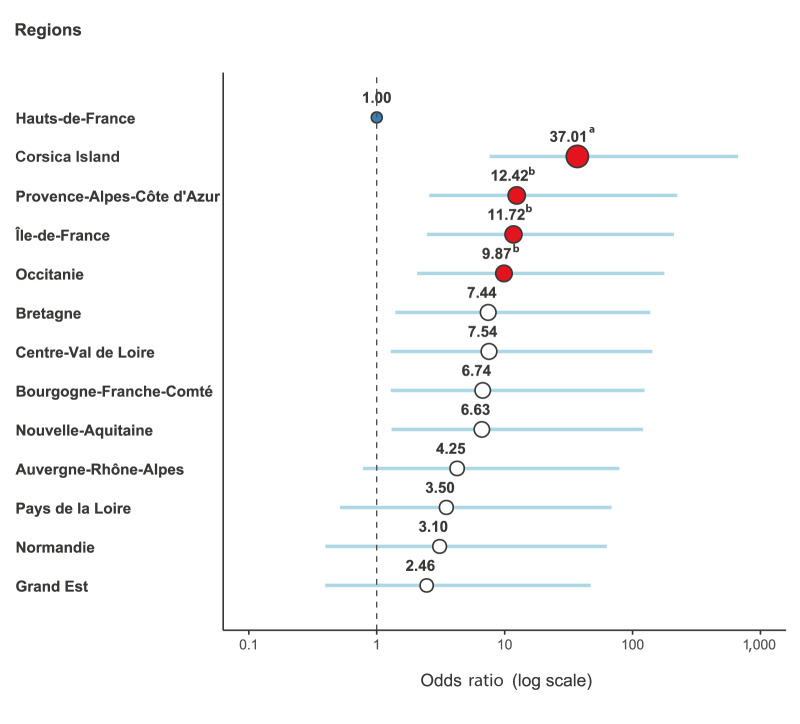
Association between region of residence and West Nile virus seropositivity, mainland France, November 2021–June 2022

Geographic residence was strongly associated with West Nile virus seropositivity, with the strongest associations observed in southern France (Corsica Island, Provence-Alpes-Côte d’Azur and Occitanie regions) and the Île-de-France region (Figure 5).

Associations are expressed as adjusted odds ratios (aOR) from a binomial logistic regression model. The size of each point is proportional to the OR magnitude. The reference region is indicated in blue. Significant associations are highlighted in red, with significance levels denoted as:

^a^ p < 0.001.

^b^ p < 0.05.

Older age was significantly associated with WNV seropositivity across the overall cohort (the association between age group and WNV seropositivity is presented in Supplementary Figure S4). A similar, though not statistically significant, trend was observed in the southern regions and the Île-de-France region.

**Figure 6:**
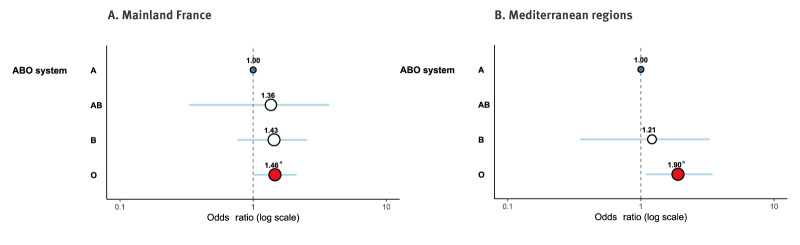
Association between ABO blood group and West Nile virus seropositivity, mainland France, November 2021–June 2022

An analysis of ABO blood groups revealed a potential association with WNV seropositivity (Figure 6; the association between each blood group and WNV seropositivity is presented in Supplementary Figure S5 and the details of positive and negative samples in the different ABO groups in France and in the southern French regions included in the data analysis are shown in Supplementary Table S5). The results suggest a potential association between ABO blood group and WNV seropositivity, with blood group O showing higher seropositivity compared with blood group A in mainland France (aOR: 1.46; 95% CI: 1.01–2.11; p value = 0.044) and in the southern regions (aOR: 1.90; 95% CI: 1.09–3.45; p value = 0.029). In mainland France, compared with other blood groups, blood group A was potentially associated with lower seropositivity (aOR: 0.69; 95% CI: 0.47–0.99; p value = 0.04).

Associations are expressed as adjusted odds ratios (aOR) from a binomial logistic regression model for the populations of (A) Mainland France and (B) Mediterranean French regions. Mediterranean regions include the Provence-Alpes-Côte d'Azur and Occitanie regions and Corsica Island. The size of each point is proportional to the magnitude of the aOR. The reference (blood group A) is indicated in blue. Significant associations are highlighted in red, with significance levels denoted as:

^a^ p < 0.05

For the Mediterranean regions, no WNV positive sample was AB positive.

Several other factors were analysed and found to have no significant association with WNV seropositivity. These included sex, the Rhesus and Kell blood group systems, living in an urban or semiurban environment or within one kilometre of a pond or river, owning domestic animals or farming poultry or horses, and a medical history of neurological conditions (meningitis, encephalitis or radiculitis).

## Discussion

This study provides important insights into the seroprevalence and circulation of WNV among blood donors in France, thereby contributing to a better understanding of its epidemiological dynamics and informing future public health strategies.

Our findings revealed a low national anti-WNV IgG seroprevalence of 0.97% among individual blood donors, consistent with previous studies from French regions. Our results align with an earlier study from the south-eastern region of Occitanie (Camargue area and Hérault department) in the early 2000s that reported a low WNV seroprevalence in humans (1.42%) living near an equine epizootic and an even lower WNV seroprevalence (0.18%) in populations living farther from the equine outbreak zone [21]. More recently (2016–2020), another study found a seroprevalence of 1.25% based on 500 human sera, which is consistent with our results [18]. Collectively, these data suggest a persistently low level of endemicity in this region, with only a modest increase in seroprevalence over the past 25 years, as indicated by the current value of 2.35% in the Hérault department using the same ELISA approach. When compared with other Mediterranean countries, our findings remain within a similar range. In Portugal, in 2022, the overall WNV seroprevalence was slightly higher (1.4%), but of the same order of magnitude as that observed in France (0.97%) [22]. In Italy, between 2008 and 2023, reported seroprevalence values ranged between 0.32% and 0.78% [23-28]. Furthermore, a study conducted among Italian blood and organ donors in 2021–2022 found a WNV seroprevalence of 0.90%, further supporting the consistency of our results with data from other southern European countries [25].

The strong correlation (R^2^ of 0.8892) between ELISA results from pooled and individual samples supports the reliability of pooled-sample testing as a cost-effective and efficient approach for large-scale serosurveys. This method can enhance surveillance efforts, particularly in resource-constrained settings. Pooled-sample testing, while more cost- and time-efficient, slightly underestimated WNV seroprevalence compared with individual testing (0.87% vs 0.97%), though the overall magnitude and geographical patterns remained consistent.

The algorithm-based confirmation method, designed to mitigate cross-reactivity with other flaviviruses such as DENV, TBEV and USUV, is intended to improve diagnostic specificity. This methodological refinement helped improve the accuracy of WNV seroprevalence estimates but could also serve as a model for harmonised flavivirus surveillance.

As anticipated, higher seroprevalence was observed in departments along the Mediterranean coast, where WNV transmission has been historically documented, notably during the 2000 outbreak in France [12] and the large 2018 outbreak across France and other European countries, with numerous cases recorded in horses, birds and humans [29]. These findings align with the established presence of competent vectors, such as *Culex pipiens*, and favourable ecological conditions (e.g., wetlands, abundant mosquitoes, suitable climate and presence of migratory bird stopover zones). Our results identified an unexpectedly high seroprevalence in regions with no prior reports of human cases, particularly Nouvelle-Aquitaine and Île-de-France. The first autochthonous cases were reported from the Gironde department (in the Nouvelle-Aquitaine region) in 2023 (29 cases) [15] and from the Île-de-France region only in 2025 [16], suggesting silent viral circulation before 2021–2022. It is also worth noting the Île-de-France region's role as a major hub with two of France's largest airports, which complicates this interpretation since travel-related exposures cannot be ruled out. No human cases were diagnosed in these regions during the study period (2021–2022), despite evidence suggesting virus circulation. This may be explained by the high proportion (around 80%) of asymptomatic infections and by limited awareness of WNV among healthcare workers at that time. Notably, equine surveillance data provided by the French West Nile Reference Laboratory (Laboratoire de Référence West Nile (LNR)) supported further evidence of local WNV circulation. Between 2020 and 2024, equine WNV cases were reported annually: seven cases in south-eastern France (Var and Corsica) in 2020–2021; nine cases in Nouvelle-Aquitaine, Provence-Alpes-Côte d’Azur and Corsica in 2022; 49 cases across the same regions, including a new detection in Gers department (Occitanie region), in 2023; and 89 cases with additional detections in Occitanie and Pays de la Loire in 2024 (personal communication, Gaëlle Gonzalez, 25 Sep 2025). These elements reinforce the hypothesis that before 2021–2022, WNV may have circulated regularly in several regions identified by our algorithm, including Nouvelle-Aquitaine.

These findings underscore the potential for undetected WNV transmission in regions or departments previously considered low risk, highlighting the need for targeted investigations to characterise transmission dynamics and anticipate potential outbreaks.

Risk factor analysis identified significant associations between WNV seropositivity and several variables. Residing in southern regions was a key risk factor, confirming previous epidemiological data and reflecting a favourable ecological environment for viral transmission (particularly mosquito abundance, warmer climates and migratory bird influxes). Interestingly, associations were observed between WNV seropositivity and ABO blood groups. Individuals with blood group O were linked to increased risk, whereas blood group A may potentially have a protective effect. The mechanism underlying this association remains unclear. Previous studies have suggested a trophic preference of certain mosquitoes for blood type O [30], but data for the *Culex* species involved in WNV transmission are lacking. The ABO blood group system may influence host immune responses, modulating antibody production or disease susceptibility. For instance, individuals with blood group O have been shown to display increased sensitivity to certain viral infections, particularly norovirus [31] and polyomavirus [32], whereas they appear less likely to develop symptomatic infection following exposure to severe acute respiratory syndrome coronavirus 2 [33-36]. Conversely, individuals with blood group A have been reported to be susceptible to HBV or HIV infection [37]. One study suggested that, for norovirus GPII.6, histological blood group antigens (HBGAs) act as receptors or attachment factors, thereby modulating individuals’ sensitivity according to their blood type and secretor status [38]. Regarding WNV, some studies have reported that individuals with blood group A are more frequently associated with symptomatic or severe cases of infection [39,40]. Since the observation of low, late or absent production of specific antibodies in neurological severe forms is not infrequent [41], our findings suggest a possible immunogenetic basis for differential host susceptibility. Given the small number of positive samples in our dataset, this hypothesis should be interpreted with caution and warrant further investigation. An age-related trend was also observed, with older individuals showing higher WNV seropositivity. This likely reflects cumulative exposure over time and is classically observed in case of exposure to endemic pathogens. Our findings are consistent with other European seroprevalence studies, including a recent large-scale survey reporting increasing WNV seroprevalence with age [42], supporting the interpretation of low-level but persistent viral circulation. In the Île-de-France region, this age-related trend, combined with elevated seroprevalence, may further support the hypothesis of silent viral circulation before 2021–2022.

While this study provides robust insights, several limitations warrant consideration. The use of blood donors provides access to a large, geographically well-distributed pool of individuals, but introduces a well-documented selection bias, mainly affecting people under the age of 18 and over the age of 70, who are excluded from donating. Blood donors are generally healthier than the general population and may not fully reflect its demographic and clinical diversity, potentially leading to under- or overestimation of the true seroprevalence. Consequently, caution is required when extrapolating absolute seroprevalence estimates to the overall population. However, the broad geographic coverage and large sample size suggest that the spatial trends observed are likely representative of those in the general population. The absence of individual travel history data limits the ability to distinguish between locally acquired and travel-related infections, particularly in regions with high population mobility. As a result, elevated seroprevalence in highly connected regions such as Île-de-France should be interpreted cautiously, as cross-sectional serology alone cannot discriminate between local circulation and past exposure acquired elsewhere. The small sample size for certain subgroup analyses, particularly for blood group associations, limits the power of the analysis. Future studies may expand sampling to better document at-risk populations and incorporate longitudinal data to track WNV circulation over time. Targeted entomological surveys in high-seroprevalence regions, such as Nouvelle-Aquitaine, and inland departments, are critical to identify vector populations and assess transmission risks.

## Conclusion

This study highlights the low but geographically variable seroprevalence of WNV in France, with evidence suggesting silent viral circulation in regions such as Nouvelle-Aquitaine and Île-de-France before reported cases. The validated use of pooled-sample ELISA testing, together with the algorithm-based confirmation approach, likely contributes to improving the precision and efficiency of WNV surveillance. These findings underscore the need for proactive public health strategies, including enhanced vector control, targeted surveillance in high-risk areas and improved blood donation screening to mitigate the risk of WNV transmission. By identifying previously unrecognised areas of WNV circulation, this study lays the groundwork for preventing future outbreaks and ensuring blood safety in France.

## Data Availability

Anonymised data are available from the corresponding author upon request.

## Supplementary Data

668000 Supplementary Material
