# Supplementary material for: Seroprevalence of West Nile virus among blood donors in mainland France, 2021 to 2022
Source: Euro Surveill. 2026 Jul 2;31(26):2500808. doi: 10.2807/1560-7917.ES.2026.31.26.2500808 (PMC13332373; doi:10.2807/1560-7917.ES.2026.31.26.2500808)
Supplement: Supplementary Material [file 25-00808_JOURDAN_Supplement.pdf]

Supplementary data

This supplementary material is hosted by *Eurosurveillance* as supporting information alongside the article "Seroprevalence of West Nile virus among blood donors in mainland France, 2021 to 2022", on behalf of the authors, who remain responsible for the accuracy and appropriateness of the content. The same standards for ethics, copyright, attributions and permissions as for the article apply. Supplements are not edited by *Eurosurveillance* and the journal is not responsible for the maintenance of any links or email addresses provided therein.

Supplementary Methods

ELISA on pooled and individual samples

Based on a 700-individual sample subset, the direct comparison of ELISA results performed on pooled samples versus individual samples demonstrated that the pooling method had identical sensitivity to individual testing (see Supplementary Figure S1).

Pooling was first performed using an Eppendorf EpMotion 5075 liquid handling system by mixing 5 µl of up to four samples together in 45 µl of ELISA's sample buffer. Then, 15 µl of the mixture was diluted in 135 µl of ELISA's sample buffer. The pool sizes were distributed as follows: 7,250 pools (54.0%) contained four samples, 3,820 (28.4%) contained three samples, 1,668 (12.4%) contained two samples, and 694 pools (5.2%) consisted of a single sample.

Virus neutralization test

Briefly, 110 µL of a serially diluted sample in DMEM with 1% of penicillin/streptomycin, Non-Essential Amino Acids and Glutamine, was mixed with 110 µL of a virus solution (0.5 $TCID_{50}$/µL of sample dilution) and incubated for 1 h at 37 °C. Subsequently, 100 µL of this mixture was transferred onto a confluent monolayer of cells (Vero E6 cells for WNV, DENV, and USUV or SW13 cells for TBEV) and incubated at 37 °C under 5% of CO_2. Cultures were assessed for cytopathic effect (CPE) seven days post-infection. The neutralisation titre was defined as the highest serum dilution showing complete neutralisation (absence of cytopathic effect).

Probability of multiple positive samples within pools

Samples were pooled in groups of up to four. Given an individual seroprevalence of 0.97% (432/44,490), the probability that a pool contains more than one positive sample was estimated using a binomial distribution. The probability that a pool of four samples contains ≥2 positives is given by:

$$P(X \geq 2) = 1 - [(1 - p)^4 + 4p(1 - p)^3],$$

where p is the individual probability of seropositivity. For $p = 0.0097$, $P(X \geq 2) \approx 5.6 \times 10^{-4}$. This corresponds to an expected frequency of fewer than 1 pool per 1,000 containing more than one positive sample. Given this low probability and the subsequent individual testing of all samples from non-negative pools, the assumption that pools contained at most one positive sample was considered reasonable and did not materially affect prevalence estimates.

Supplementary figures

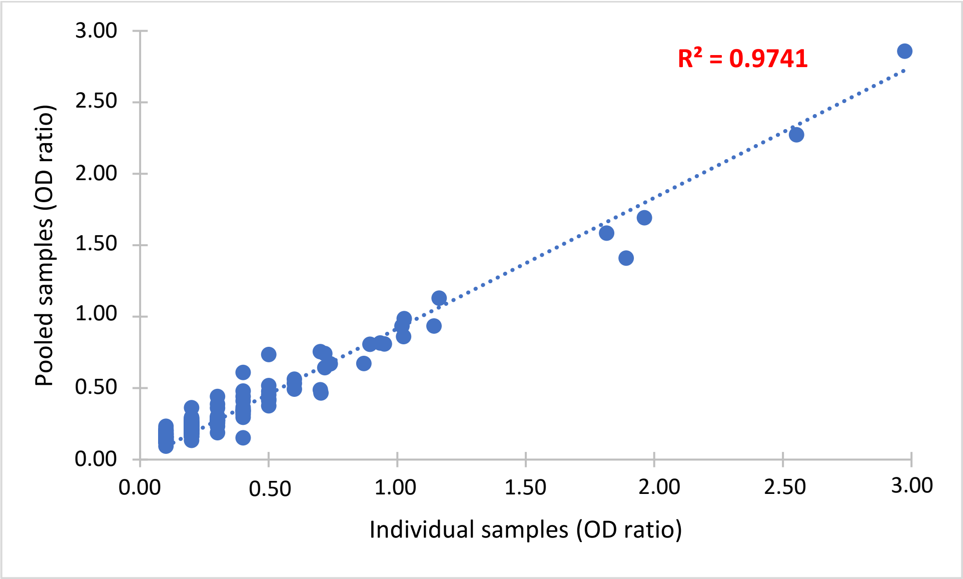

Supplementary Figure S1. Correlation between the ELISA ratio of pooled and individual samples.

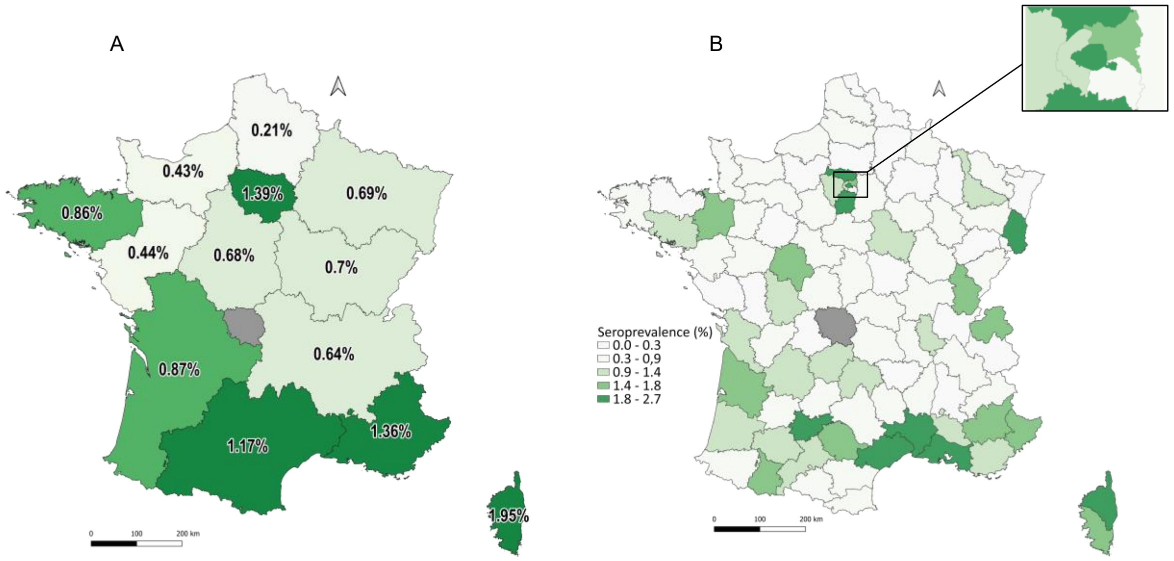

Supplementary Figure S2. maps of anti-WNV IgG positive pooled samples seroprevalence, in administrative regions (A), in departments (B).

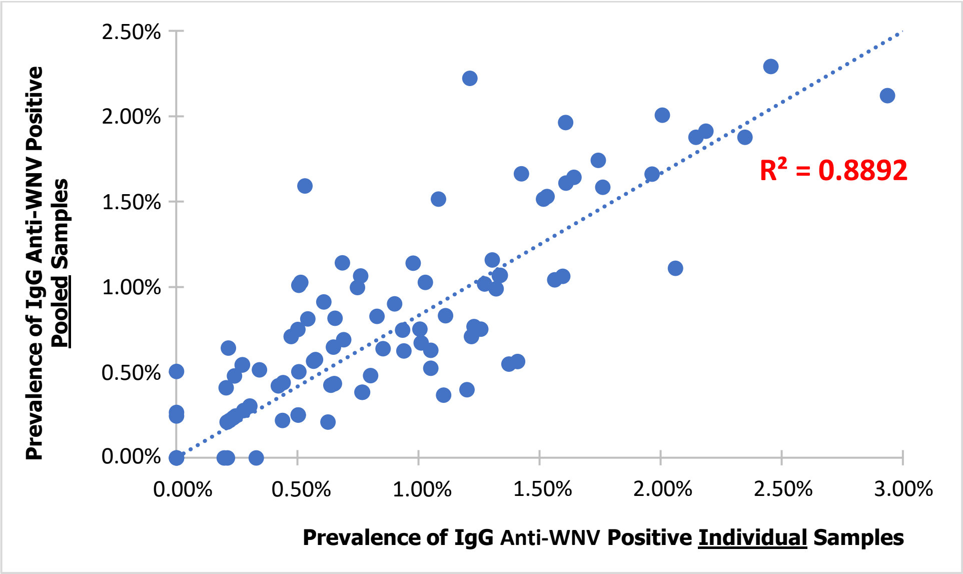

Supplementary Figure S3. Correlation between WNV seroprevalences obtained from pooled and individual samples at the department level.
Each point represents the prevalence from a department calculated from the ELISA results.

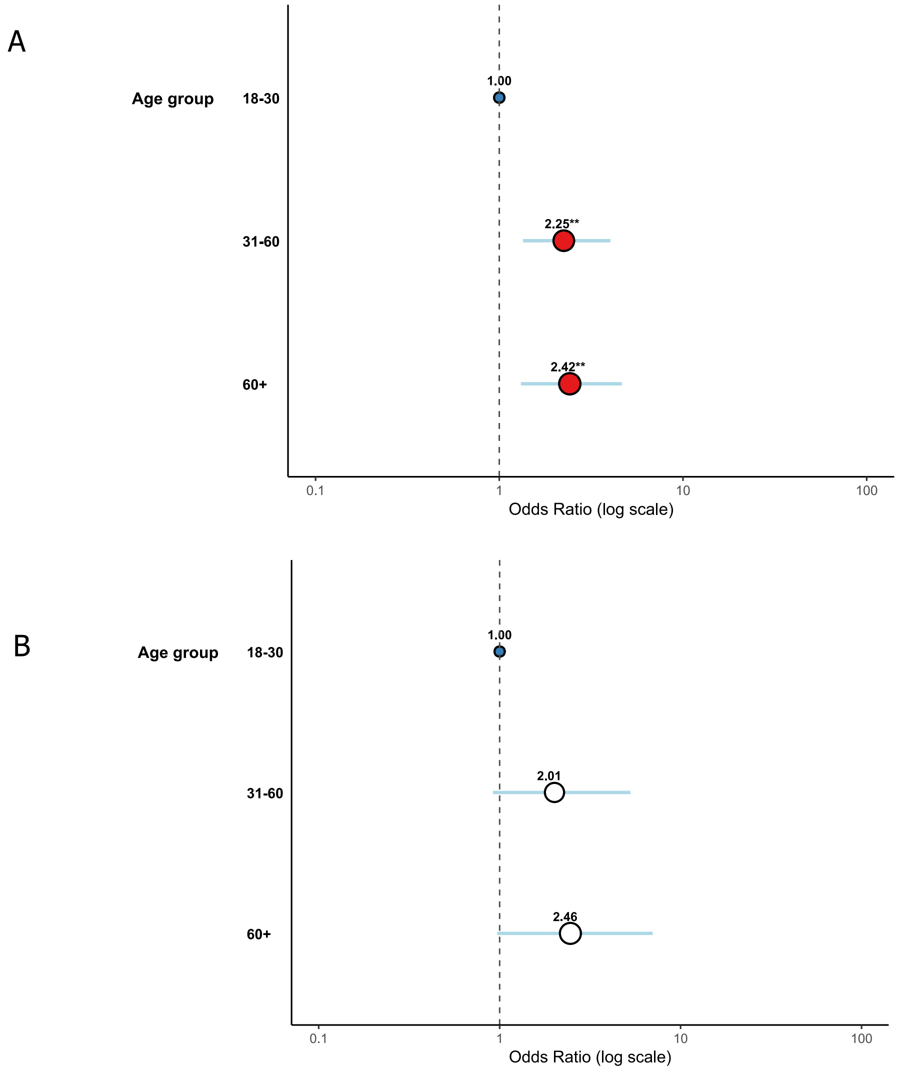

C

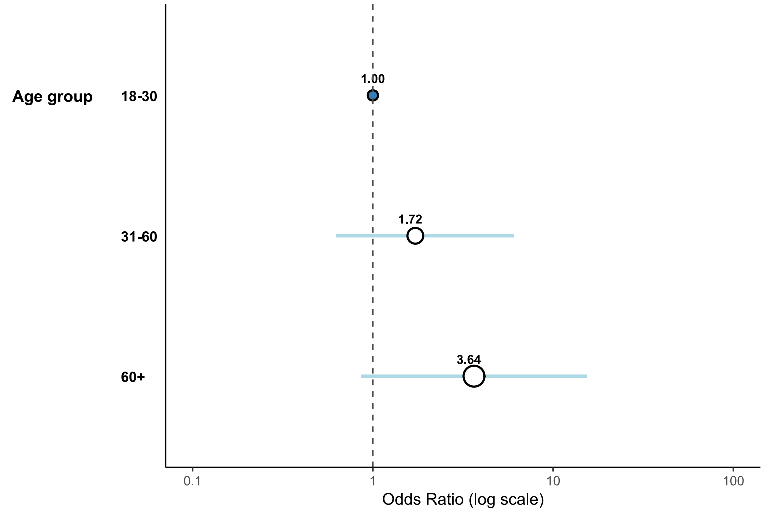

Supplementary Figure S4. Association between Age group and WNV seropositivity.

Associations are expressed as odd ratios (OR) from a multivariate binomial logistic regression model using algorithm-defined WNV serostatus (confirmed + probable as positives) for the populations of France (A), Southern French regions (B), Île-de-France region (C). The size of each point is proportional to the OR magnitude. The reference region is indicated in blue. Significant associations are highlighted in red, with significance levels denoted as: *p<0.05, **p<0.01, ***p<0.001. This analysis was exploratory.

A

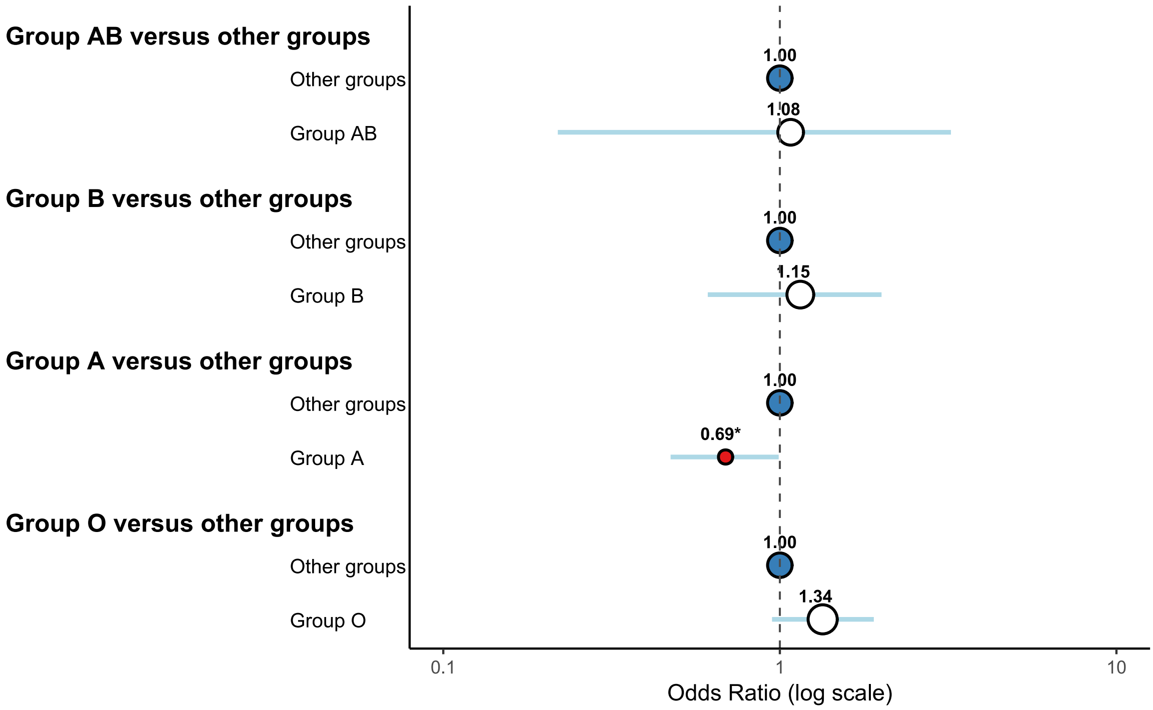

B

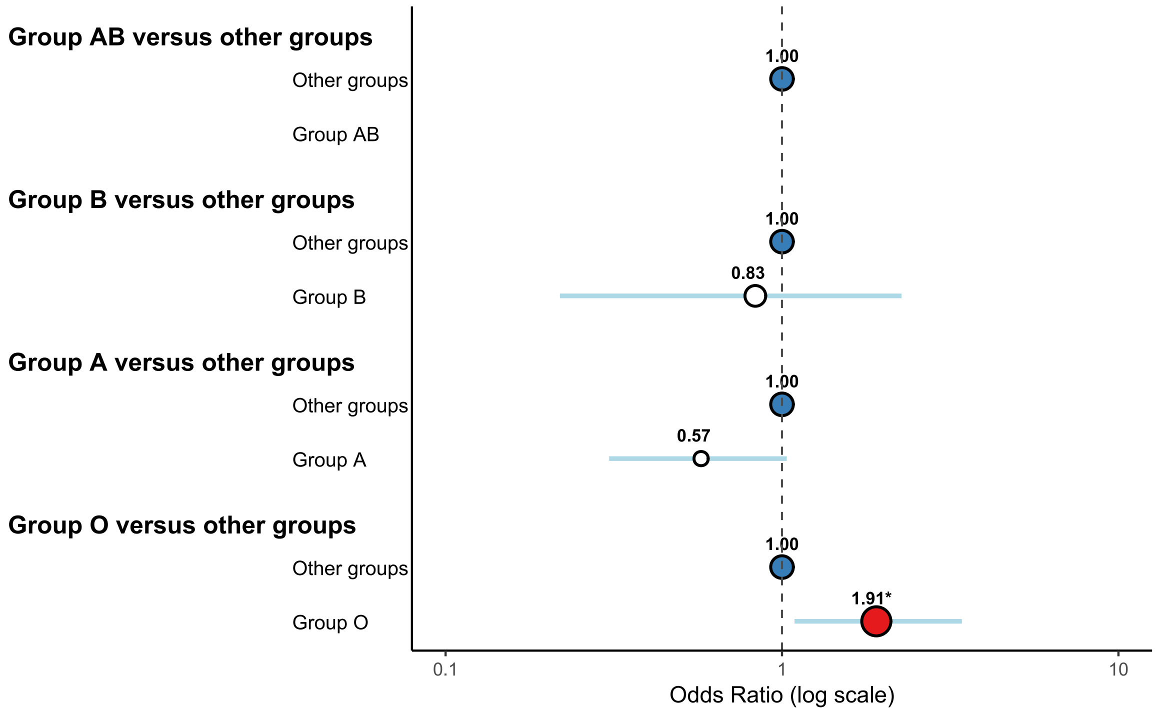

Supplementary Figure S5. Association between blood groups and WNV seropositivity.

Associations are expressed as odd ratios (OR) from a univariate Fisher's exact test, using algorithm-defined WNV serostatus (confirmed + probable as positives), for the populations of France (A), Southern French regions (B). The size of each point is proportional to the OR magnitude. The reference region is indicated in blue. Significant associations are highlighted in red, with significance levels denoted as: *p<0.05, **p<0.01, ***p<0.001. Results should be interpreted cautiously given the limited number of positive samples and are considered exploratory.

Supplementary tables

Supplementary Table S1. Numbers of samples by department and administrative regions.

Administrative regions	Number of samples	Departments	Number of samples
Auvergne-Rhône-Alpes	4,559	**Ain**	**409**
		**Allier**	**381**
		**Ardèche**	**297**
		**Cantal**	**396**
		**Drôme**	**377**
		**Haute-Loire**	**372**
		**Haute-Savoie**	**377**
		**Isère**	**399**
		**Loire**	**395**
		**Puy-de-Dôme**	**369**
		**Rhône**	**389**

		**Savoie**	**398**
Hauts-de-France	2,379	**Aisne**	**477**
		**Nord**	**475**
		**Oise**	**464**
		**Pas-de-Calais**	**488**
		**Somme**	**475**
Provence-Alpes-Côte d'Azur	3,686	**Alpes-de-Haute-Provence**	**574**
		**Alpes-Maritimes**	**609**
		**Bouches-du-Rhône**	**560**
		**Var**	**690**
		**Hautes-Alpes**	**639**
		**Vaucluse**	**614**
Grand Est	4,955	**Ardennes**	**417**
		**Aube**	**454**
		**Bas-Rhin**	**479**
		**Marne**	**506**
		**Haute-Marne**	**555**
		**Haut-Rhin**	**495**
		**Meurthe-et-Moselle**	**657**
		**Meuse**	**457**
		**Moselle**	**469**
		**Vosges**	**466**
Occitanie	4,887	**Ariège**	**368**
		**Aude**	**353**
		**Aveyron**	**367**
		**Lot**	**360**
		**Gard**	**373**
		**Gers**	**374**
		**Haute-Garonne**	**389**
		**Hautes-Pyrénées**	**421**
		**Hérault**	**426**
		**Lozère**	**362**
		**Pyrénées-Orientales**	**355**
		**Tarn**	**373**
		**Tarn-et-Garonne**	**366**
Normandie	2,311	**Calvados**	**463**
		**Eure**	**455**
		**Manche**	**463**
		**Orne**	**460**
		**Seine-Maritime**	**470**
Nouvelle-Aquitaine	4,351	**Corrèze**	**401**
		**Charente**	**422**
		**Charente-Maritime**	**375**
		**Deux-Sèvres**	**398**
		**Dordogne**	**376**

		**Gironde**	**397**
		**Haute-Vienne**	**409**
		**Landes**	**384**
		**Lot-et-Garonne**	**398**
		**Pyrénées-Atlantiques**	**398**
		**Vienne**	**393**
Centre-Val de Loire	1,911	**Cher**	**250**
		**Eure-et-Loir**	**272**
		**Indre**	**260**
		**Indre-et-Loire**	**392**
		**Loiret**	**476**
		**Loir-et-Cher**	**261**
Bourgogne-Franche-Comté	3,877	**Côte-d'Or**	**624**
		**Jura**	**396**
		**Doubs**	**611**
		**Haute-Saône**	**319**
		**Nièvre**	**360**
		**Saône-et-Loire**	**594**
		**Territoire de Belfort**	**535**
		**Yonne**	**438**
Bretagne	2,911	**Cotes-d'Armor**	**607**
		**Finistère**	**985**
		**Ille-et-Vilaine**	**662**
		**Morbihan**	**657**
Pays de la Loire	2,728	**Loire-Atlantique**	**696**
		**Maine-et-Loire**	**583**
		**Mayenne**	**364**
		**Sarthe**	**426**
		**Vendée**	**659**
Ile-de-France	4,756	**Paris**	**616**
		**Essonne**	**598**
		**Hauts-de-Seine**	**631**
		**Seine-et-Marne**	**579**
		**Seine-Saint-Denis**	**462**
		**Val-de-Marne**	**651**
		**Val-d'Oise**	**613**
		**Yvelines**	**606**
Corse	1,179	**Corse-du-Sud**	**568**
		**Haute-Corse**	**611**
**Total number of samples**	**44,490**		

Classified as ECDC NORMAL

Supplementary Table S2. Socio-demographic data collected along to the blood sample used within this study.

Socio-demographic data	Questions	Possible answers
**Sex**		
**Blood group ABO system**		
**Rhesus status**		
**Kell status**		
**Regions/departments of residence**		
**Living in an urban or semi-urban environment**	Distance from the place of residence to the nearest supermarket	Less than 15 minutes by car / Between 15 and 30 minutes by car / More than 30 minutes by car / I don't know
	Distance to the nearest shop (grocery / café-tobacco / bakery) from the place of residence	Less than a minute on foot / 1 to 5 minutes on foot / 5 to 10 minutes on foot / More than 10 minutes on foot / I don't know
	Do you live less than one kilometer from ponds or marshes?	Yes / No
	Do you live less than one kilometer from rivers?	Yes / No
**Domestic animal ownership**	Do you have any pets?	Yes / No
	If yes / which are those animals? Are they living inside or outside the house?	Inside: possible choices: Cats / dogs / horses / rodents / birds / others
		Outside: possible choices: Cats / dogs / horses / rodents / birds / others
**Breeding**	Do you practice animal breeding?	No / Yes / choices: ducks-gooses / cows / goats / bunnies / chickens / pigs / sheeps / Horses-ponies / others
**Medical history of neurological diseases**	Have you ever had meningitis / encephalitis / meningoencephalitis / or radiculitis?	Yes / No / I don't know
	If yes / please specify the condition	meningitis / encephalitis / meningoencephalitis / radiculitis / I don't know / other

All variables listed were initially explored in univariate analyses; a predefined subset of epidemiologically relevant variables was included a priori in the multivariate models, as detailed in the Methods.

Classified as ECDC NORMAL

Supplementary Table S3. Details of the different algorithm categories.

140 samples were classified as confirmed/probable positives.

Algorithm categories	Criteria	Number (%)	
CONF1	WNV VNT titre is >10 and twice as high as the VNT titres of TBEV, DENV, USUV	16 (0.04%)	Confirmed and Probable WNV: 0.31%
CONF2	Not « CONF1 » and WNV VNT titre >10 and VNT titres of TBEV, DENV, USUV =10	5 (0.01%)	
CONF3	Not « CONF1 or CONF2 » and with WNV ELISA ratio >1.1 and twice as high as the ELISA ratio of TBEV, DENV, USUV, and a WNV VNT titre >10 and ≥ the VNT titres of TBEV, DENV, USUV	0 (0%)	
Probable	Not « CONF1, 2 or 3 », and with a WNV ELISA ratio >1.1 and > the ELISA ratio of TBEV, DENV, USUV and a WNV VNT titre ≥ the VNT titres of TBEV, DENV, USUV	119 (0.27%)	
Possible	no other status can apply and WNV ratio is ≥0.8, and the 0.75*WNV ratio is > the ELISA ratio of TBEV, DENV, USUV	6 (0.01%)	Possible WNV or Flavivirus: 0.84%
Other	Samples that do not belong to any other status but with WNV ELISA ratio >0.8 (presumably mostly samples with antibodies against other flaviviruses)	365 (0.82%)	
Negative (NEG)	WNV ELISA ratio <0.8 and a WNV VNT titre=10 (*i.e.*, negative)	43,979 (98.85%)	Negative: 98.85%
**Total**		**44,490 (100%)**	

Supplementary Table S4. Details of the different seroprevalences (anti-WNV IgG ELISA vs Algorithm) with the confidence interval 95% in the French regions

Regions	Pool-based seroprevalence	Anti-WNV IgG ELISA seroprevalence	Algorithm-based seroprevalence
Auvergne-Rhône-Alpes	0.64% (95% CI: 0.43%-0.91%)	0.42% (95% CI: 0.25%-0.65%)	0.18% (95% CI: 0.08%-0.35%)
Hauts-de-France	0.21% (95% CI: 0.07%-0.49%)	0.17% (95% CI: 0.05%-0.43%)	0.04% (95% CI: 0.00%-0.23%)
Provence-Alpes-Côte d'Azur	1.36% (95% CI: 1.01%-1.78%)	1.36% (95% CI: 1.01%-1.78%)	0.52% (95% CI: 0.31%-0.80%)
Grand Est	0.69% (95% CI: 0.48%-0.96%)	0.59% (95% CI: 0.39%-0.84%)	0.10% (95% CI: 0.03%-0.24%)
Occitanie	1.17% (95% CI: 0.88%-1.51%)	1.29% (95% CI: 0.99%-1.65%)	0.45% (95% CI: 0.28%-0.68%)
Normandie	0.43% (95% CI: 0.21%-0.79%)	0.56% (95% CI: 0.30%-0.96%)	0.13% (95% CI: 0.03%-0.38%)
Nouvelle-Aquitaine	0.87% (95% CI: 0.62%-1.20%)	1.13% (95% CI: 0.83%-1.49%)	0.28% (95% CI: 0.14%-0.48%)
Centre-Val de Loire	0.68% (95% CI: 0.36%-1.16%)	1.10% (95% CI: 0.68%-1.67%)	0.31% (95% CI: 0.12%-0.68%)
Bourgogne-Franche-Comté	0.70% (95% CI: 0.46%-1.01%)	0.70% (95% CI: 0.46%-1.01%)	0.26% (95% CI: 0.12%-0.47%)
Bretagne	0.86% (95% CI: 0.56%-1.27%)	1.10% (95% CI: 0.75%-1.55%)	0.31% (95% CI: 0.14%-0.59%)
Pays de la Loire	0.44% (95% CI: 0.23%-0.77%)	0.51% (95% CI: 0.28%-0.86%)	0.15% (95% CI: 0.04%-0.37%)
Ile-de-France	1.39% (95% CI: 1.07%-1.76%)	1.81% (95% CI: 1.45%-2.23%)	0.48% (95% CI: 0.31%-0.72%)
Corse	1.95% (95% CI: 1.24%-2.91%)	2.12% (95% CI: 1.38%-3.11%)	1.53% (95% CI: 0.91%-2.40%)

95% CI : confidence interval 95%

Classified as ECDC NORMAL

Supplementary Table S5. Details of the different ABO groups in France and in the southern French regions included in the data analysis

ABO groups	France		Southern French regions	
	WNV Negative	WNV Positive	WNV Negative	WNV Positive
**A group**	18,255 (41.03%)	46 (0.10%)	4,159 (9.35%)	17 (0.04%)
**B group**	3,872 (8.93%)	14 (0.03%)	809 (1.82%)	4 (0.009)
**AB group**	876 (1.97%)	3 (0.007%)	197 (0.44%)	0 (0%)
**O group**	20,975 (47.14%)	77 (0.17%)	4,900 (11.01%)	38 (0.085%)

Positives come from the sum of Confirmed and Probable samples
